# Diet and high altitude strongly drive convergent adaptation of gut microbiota in wild macaques, humans, and dogs to high altitude environments

**DOI:** 10.3389/fmicb.2023.1067240

**Published:** 2023-02-23

**Authors:** Junsong Zhao, Yongfang Yao, Mengmeng Dong, Hongtao Xiao, Ying Xiong, Shengzhi Yang, Diyan Li, Meng Xie, Qingyong Ni, Mingwang Zhang, Huailiang Xu

**Affiliations:** ^1^College of Life Science, Sichuan Agricultural University, Ya’an, China; ^2^College of Agronomy and Life Sciences, Zhaotong University, Zhaotong, China; ^3^School of Pharmacy, Chengdu University, Chengdu, China; ^4^College of Animal Science and Technology, Sichuan Agricultural University, Chengdu, China

**Keywords:** high altitude environment, convergent adaptation, gut microbiota, 16S rRNA gene, rhesus macaque

## Abstract

Animal gut microbiota plays an indispensable role in host adaptation to different altitude environments. At present, little is known about the mechanism of animal gut microbiota in host adaptation to high altitude environments. Here, we selected wild macaques, humans, and dogs with different levels of kinship and intimate relationships in high altitude and low altitude environments, and analyzed the response of their gut microbiota to the host diet and altitude environments. Alpha diversity analysis found that at high altitude, the gut microbiota diversity of wild macaques with more complex diet in the wild environments is much higher than that of humans and dogs with simpler diet (*p* < 0.05), and beta diversity analysis found that the UniFrac distance between humans and dogs was significantly lower than between humans and macaques (*p* < 0.05), indicating that diet strongly drive the convergence of gut microbiota among species. Meanwhile, alpha diversity analysis found that among three subjects, the gut microbiota diversity of high altitude population is higher than that of low altitude population (ACE index in three species, Shannon index in dog and macaque and Simpson index in dog, *p* < 0.05), and beta diversity analysis found that the UniFrac distances among the three subjects in the high altitude environments were significantly lower than in the low altitude environments (*p* < 0.05). Additionally, core shared ASVs analysis found that among three subjects, the number of core microbiota in high altitude environments is higher than in low altitude environments, up to 5.34 times (1,105/207), and the proportion and relative abundance of the core bacteria types in each species were significantly higher in high altitude environments than in low altitude environments (*p* < 0.05). The results showed that high altitude environments played an important role in driving the convergence of gut microbiota among species. Furthermore, the neutral community model trial found that the gut microbiota of the three subjects was dispersed much more at high altitude than at low altitude, implying that the gut microbiota convergence of animals at high altitudes may be partly due to the microbial transmission between hosts mediated by human activities.

## 1. Introduction

The structure of gut microbiota is the result of the interaction and coevolution of the host, as well as environmental factors (Blaut et al., 2002). Animal genetic relationships (Goodrich et al., 2014), dietary (De Filippo et al., 2010; Goodrich et al., 2014), altitude (Zhao et al., 2018; Zeng et al., 2020), season (Xia et al., 2021), and other environmental factors have a profound impact on the composition and structure of gut microbiota. The diet structure of the host directly influences the gut microbiota (De Filippo et al., 2010; Huang et al., 2022), and similar diets drive the convergence evolution of gut microbiota in animals (Huang et al., 2021). The gut microbiota of pandas (*Ailuropoda melanoleuca*) and red pandas (*Ailurus fulgens*) differ significantly from those of other species in the Carnivora order, but they share a similar core gut microbiota as insects consuming bamboo, indicating that their diet is a major driving force for the convergence of gut microbiota in these species (Yao et al., 2021). However, other studies also found that the composition of the gut microbiota of giant pandas is more similar to that of bears and completely different from that of other herbivores, with a low level of cellulose digesting bacteria (Xue et al., 2015). Further research has confirmed that the gut microbiota of giant pandas cannot adapt well to the degradation of cellulose and lignin in the high-fiber bamboo diet, but has evolved to utilize more digestible carbohydrates to maximize the intake of nutrients and energy from bamboo (Zhang et al., 2018). Through the fecal microbial transmission (FMT) of germ-free (GF) mice, it was also found that in the first few days after FMT, the difference of gut microbiota among GF mice with different donor microbiota would decrease, but when the gut microbiota was stable, the difference would increase, which proved that the gut microbiota had greater impact than diet (Zhang et al., 2022). These studies show that diet has limited influence on gut microbiota composition of giant pandas.

Meanwhile, in vertebrates, gut microbiota compositional differences among species are positively correlated with the evolutionary divergence time of the host, and the gut microbiota composition is more similar within host species than among species (Moeller et al., 2017; Song et al., 2020; Dillard et al., 2022). Additionally, environmental factors have been confirmed to be closely related to the gut microbiota structure (Ley et al., 2008; Zhao et al., 2018) and the extreme cold, the dry, hostile climate of high altitude environments, high ultraviolet radiation, and low oxygen content have important effects on the cardiovascular system, energy metabolism, and body temperature retention of animals (Simonson et al., 2010; Yu et al., 2016; Zhu et al., 2021). A variety of mammalian gut microbiota also respond to this environmental pressure, forming a composition of gut microbiota that adapts to the high altitude environments, and play an important role in host food digestion, energy metabolism, nutritional homeostasis, immune regulation, signal transduction, and other physiological activities (Bäckhed et al., 2005; Ley et al., 2006; Yan et al., 2021). Studies on humans (Li and Zhao, 2015; Li K. et al., 2016), macaques (Zhao et al., 2018; Wu et al., 2020), pigs (Zeng et al., 2020), and other mammals (Li H. et al., 2016) showed that altitude differences in environmental factors have an important influence on gut microbiota composition. The high altitude rumen microbiota of yak and Tibetan sheep exhibited a convergent phenomenon, with significantly lower levels in production of methane and volatile fatty acids (VFAs) (Zhang et al., 2016). The high altitude environments drive the diversity of gut microbiota composition and convergent evolution of indicator microbiota in ungulates (Wang et al., 2022). Studies on a variety of ungulates living in high altitude environments, such as the Tibetan antelope (*Pantholops hodgsoni*) and Tibetan sheep, also found that they have a similar composition of gut microbiota (Ma et al., 2019). These studies fully illustrate that high altitude, extreme environments have important driving effects on the compositional structure of the gut microbiota of animals.

The animals gut microbiota are shaped by the dispersal of organisms into habitats, followed by natural selection (i.e., habitat filtration), drift, and *in situ* diversification (Vellend, 2010). Studies have found that the mammalian microbiota is acquired vertically from mother to offspring (Dominguez-Bello et al., 2010; Vaishampayan et al., 2010) through genetic effects and horizontally among non-relatives through social interactions and shared environments (Tung et al., 2015; Moeller et al., 2016). The contact between host species results in the widespread dissemination of bacteria and homogenisation of microbial communities within mammalian communities (Moeller et al., 2017). In addition, there are routes of gut microbiota transmission between distantly related vertebrate species through humans and urban environments (Dillard et al., 2022), and the wildlife gut microbiota in its urban environment is also gradually humanized (Dillard et al., 2022). The gut microbiota composition of hosts is influenced by migratory dispersal among different species and that activity patterns in humans also drive the humanization of gut microbiota composition in wildlife, which may have important consequences for the host phenotype and environmental fitness (Fackelmann et al., 2021; Dillard et al., 2022), however, the natural resource-dependent lifestyle of human beings in high altitude areas is mainly based on grazing and collection under forests, which means they have a high temporal and spatial overlap with wild animals in the area. Little is known about the impact of gut microbiota community composition of wild and domestic animals, in particular, the degree of integration of wild animals, domestic animals, and the human gut microbiota in the extreme environment at high altitudes has not been fully explored.

Studies on phylogenetic relationships showed that dogs are different from humans and monkeys about 85 million years ago, while humans differed from monkeys about 23.5–34 Ma ago (Dos Reis et al., 2012). Meanwhile, rhesus macaques have high homology with humans in morphology, physiology, biochemistry, genetics, and reproduction (Chan et al., 2001) and are also one of the most widely distributed animals in the natural environment. Dogs, as important companion animals for humans, were domesticated approximately 40,000 to 14,000 years ago and have a more similar diet to humans and close contact with (Wang et al., 2013, 2016). They are also one of the animals more deeply influenced by human activities, the typical representative animals inhabited cultural environments. Therefore, wild macaques, humans, and dogs are ideal for evaluating the effects of diet and altitude environments on the hosts gut microbiota. Here, we compared the gut microbiota composition of humans, dogs and wild macaques housed at high altitude (altitude >3,000 m) and low altitude (altitude <1,000 m) environments, revealed the effects of diet and altitude environments on the host gut microbiota, and assessed the effects of extreme conditions at high altitude on the gut microbiota of wild macaques, humans, and dogs. The results are important for understanding the mechanism of environmental adaptation to high altitude for humans and animals, as well as for the conservation of wildlife, domestic animal feeding, and guiding the rational use of natural resources by humans.

## 2. Materials and methods

### 2.1. Ethics statement

Before sample collection, all the animal work was approved by the Animal Welfare and Animal Ethics Committee of Sichuan Agricultural University (SKY-S20171006). The human samples and the relevant information were kept confidential. All fieldwork was granted permission by the Administration of Wild Animal and Plant Protection, Nature Reserves, The Department of Forestry in Tibet provincial region and Chongqing provinces.

### 2.2. Faecal sample collection

**Figure 1:**
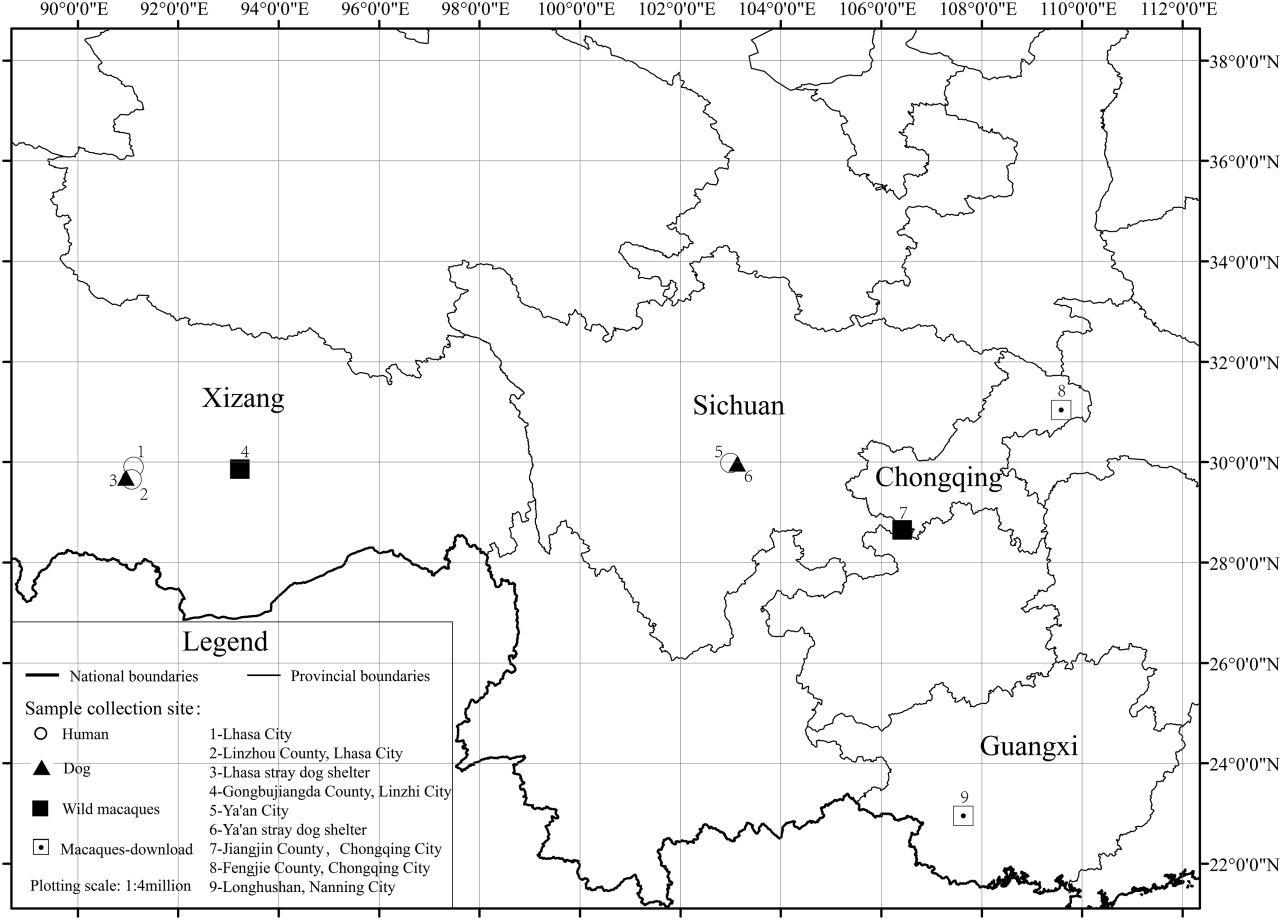
Sample collection profile.

Due to the genetic relationship between coyotes and dogs, we also downloaded 18 coyote data at low altitudes for comparative analysis. A total of 152 fecal samples (40 human, 40 dog, 54 wild macaque, and 18 coyote) were enrolled in our diversity study of gut microbiota through a 16S rRNA gene V3–V4 high-throughput sequencing approach. One hundred and nine samples were newly collected in this study, and the data from 25 macaques and 18 coyotes at low altitude were retrieved from previously published studies (SequenceRead Archive number: PRJNA535368, PRJNA528764, and PRJNA528765) (Sugden et al., 2020, 2021; Wu et al., 2020). A total of 7 groups were divided according to altitude and animal species. The number of newly collected samples was determined based on the number of individuals from most groups of macaques, which, through our previous knowledge, had been found to consist mostly of 40–50 individuals, including a certain number of juvenile individuals. Therefore, we planned to collect a sample size of 10–20 adult individuals per population, with the number of samples from humans and dogs also determined with reference to the number of samples from wild macaques. Among them, wild macaque samples were collected from wild populations, including those in Linzhi County of Tibet and Jiangjin County of Chongqing. The dog samples were collected at a Tibetan stray dog shelter and a Ya’an stray dog shelter. Human samples were collected from Linzhou County, Lhasa, Tibet, and Ya’an City, Sichuan Province (Table 1; Figure 1). Through our observation, we found that humans and dogs in Tibet eat a lot of high-protein food every day, such as butter, yak meat, and highland barley, compared with low altitudes in Ya’an City, Sichuan Province, the intake of plant fiber is relatively small. Meanwhile, in high altitude areas, due to the selection pressure of the extreme environments, the human lifestyle is mainly based on natural resources, such as cutting cordyceps under the forest, mushrooms, and grazing yaks, which increases the spatial and temporal overlap with wild animals. In addition, we collected human fecal samples from two different places at the same altitude, and there is no direct kinship between these humans. There is no direct kinship in the same species between high altitude and low altitude. Moreover, the two sampling sites are far away from each other, and there is no close contact between the sampling population. For rhesus macaques sample collection, we choose a continuous period of time in a day to follow the rhesus macaques and take samples through direct observation. Samples with a distance greater than 5 meters are recorded as samples from different individuals. We followed groups of rhesus macaques for as much fresh sample collection as possible in an hour, while guaranteeing that no fewer than 20 fresh samples were collected per population. For the downloaded macaques data, DNA extraction methods, sequencing methods and primers are consistent with this study. Therefore, we have not pursued a comparative discussion. Fecal samples were collected with sterile gloves, put into the sampling box at −20°C, and brought back to the laboratory for storage at −80°C.

**Table 1:** Sampling information table.

Group	Sample number	Sex	Age	Site	Altitude	Sample source
	HH1	Female	21				HH2	Female	21	HH3	Female	21	HH4	Female	20	HH5	Female	21	Lhasa City, Tibet	3650	Fecal	HH6	Male	21	HH7	Male	22				HH8	Male	22				HH9	Male	21			
High altitude Human (HH)	HH10	Male	21																		
	HH11	Female	46				HH12	Female	41				HH13	Female	38	HH14	Female	46	HH15	Female	45	Linzhou County, Lhasa City, Tibet	3900	Fecal	HH16	Male	53	HH17	Male	37				HH18	Male	28	HH19	Male	34	HH20	Male	44
High altitude Dog (HD)	HD1-20	—	—	Lhasa stray dog shelter	3650	Fecal																		
High altitude Rhesus macaques (HM)	HM1-19	—	Adult	Gongbujiangda County, Linzhi City	3400	Fecal
	LH1	Female	25				LH2	Female	24	LH3	Female	25	LH4	Female	22	LH5	Female	22	LH6	Male	25	LH7	Male	24	LH8	Male	24	LH9	Male	26
Low altitude Human (LH)	LH10	Male	29	Ya’an, Sichuan	560	Fecal
	LH11	Female	44			
	LH12	Female	47				LH13	Female	46	LH14	Female	45	LH15	Female	46	LH16	Male	46	LH17	Male	47	LH18	Male	48	LH19	Male	45	LH20	Male	47
Low altitude Dog (LD)	LD1-20	—	—	Ya’an stray dog shelter	590	Fecal
Low altitude Rhesus macaques (LM)	LM1-10	—	Adult	Jiangjin County, Chongqing City	895	Fecal
	LM11-20	—	Adult	Fengjie County, Chongqing City	220	PRJNA535368	LM21-35	—	Adult	Longhushan, Guangxi	220	PRJNA535368
Low altitude Coyote (LW)	LM1-18	—	—	Dround Edmonton, Alberta, Canada	-	PRJNA528764
						PRJNA528765

### 2.3. DNA extraction and PCR amplification

Microbial genomic DNA was extracted from fecal samples using a TIANamp Stool DNA kit (Tiangen, Beijing, China). The integrity of the extracted genomic DNA was verified by 1.0% agarose gel electrophoresis. The V3-V4 regions of the bacterial 16S rRNA gene (from 341 to 806) were amplified from extracted DNA using the barcoded primers 341\u00B0F (5′- CCTACGGGNGGCWGCAG −3′) and 806 R (5′ GGACTACNVGGGTATCTAAT-3′) (Fadrosh et al., 2014), with a Biometra TOne 96 G PCR thermocycler (Germany). PCR amplification of the 16S rRNA gene was performed as previously described in Wu et al. (2020) (Wu et al., 2020). Specifically, the PCR was performed in a 50-μL reaction system containing 1.5 μL of each primer, 100 ng template DNA, 5 μL 10 × KOD Buffer, 5 μL 2.5 mM dNTPs, and 1 μL KOD polymerase. The PCR conditions consisted of a denaturation step at 95°C for 2 min, and amplification was carried out with 27 cycles at a melting temperature of 98°C for 10 s, an annealing temperature of 62°C for 30 s, and an extension temperature of 68°C for 30 s. The final extension step was performed at 68°C for 10 min. The barcoded PCR products were purified using a DNA gel extraction kit (Axygen, China) and quantified using Quantus™ Fluorometer (Promega, USA) (Wu et al., 2020). The purified amplicons were pooled in equimolar amounts and paired-end sequenced on an Illumina Hiseq PE250 platform, according to the standard protocols by Genedenovo Inc. (Guangzhou, China).

### 2.4. Processing of sequencing data

Because the downloaded Coyote data were v4–v5 regions, we used USEARCH for tiling alignments after processing the sequences, and then used the plug-in “cutadapt” of QIIME2 to remove paired end reads from the primers and truncate the V4 region for subsequent analysis (Hall and Beiko, 2018). The plug-in “DADA2” was used to control sequence quality, correct amplicon errors, and generate ASVs (Callahan et al., 2016). Chimeras were filtered and the ASVs present in at least 2 samples were retained. Based on Silva_ 132 databases, trained a classification classifier against the bacterial V4 region of the 16S rRNA gene, and used this classifier to generate a classification map of out data. The resulting alignment was used for subsequent statistical analysis. Furthermore, the beta diversity distance matrices of the microbial community were calculated and performed by QIIME2.

### 2.5. Statistical analysis

The alpha diversity Shannon index, ACE index, Simpson index, weighted and unweighted UniFrac distances were calculated by Qiime 2, and the Statistics significance test for each group in R statistical software (version 4.1.3). Linear Discriminant Analysis (LDA) Effect Size (LEfSe) was analyzed and visualized through Galaxy online platform. Venn (VN) map analysis and visualization were done *via* the online platform EVenn (Chen et al., 2021). Principal Co-ordinates Analysis (PCoA) and neutral community model (NCM) analysis were done by R (Chen et al., 2019), and part of the results visualization was done by the online platform ImageGP (Chen T. et al., 2022).

## 3. Results

### 3.1. Multivariate statistical analysis of gut microbiota diversity

**Figure 2:**
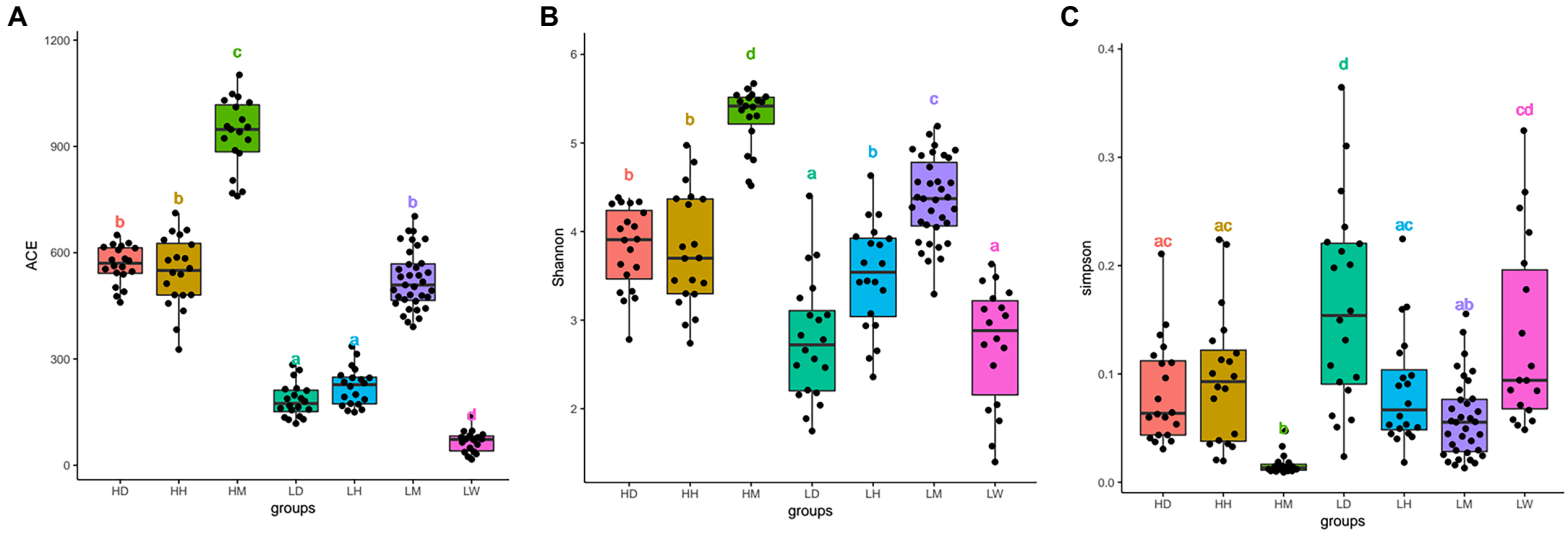
Microbiota alpha diversity analysis of fecal samples among wild macaques, humans and dogs in high altitude and low altitude environments. The alpha diversity among different groups **(A)** ACE index; **(B)** Shannon diversity; **(C)** Simpson diversity. The same letter indicates the difference is not significant (T-test, *p* > 0.05). HH stands for high altitude humans, HD stands for high altitude dogs, HM stands for high altitude wild macaques, LH stands for low altitude humans, LD stands for low altitude dogs, LM stands for low altitude wild macaques, LW stands for low Coyote.

After quality filtering, we obtained 14,331,096 raw reads across 152 fecal samples. The sequences were clustered at 100% sequence identity and 4,320 Amplicon sequence variants (ASVs) were generated. After dilution flattening by the minimum number of sequences, the ACE index (mean ± SD, 445 ± 269), Shannon index (mean ± SD, 3.81 ± 0.97), and Simpson index (mean ± SD, 0.09 ± 0.07) were used to assess the gut microbiota alpha diversity (Figures 2A–C; Supplementary Table S1). The results showed that the gut microbiota diversity of wild macaques in the same altitude environments was significantly higher than that of other species (*p* < 0.05). There was no significant difference in the diversity between humans and dogs in the high altitude environments, and there was no significant difference in the ACE index between humans and dogs in the low altitude population, however, the Shannon and Simpson indexes showed that the diversity of humans was significantly higher than that of dogs. A comparison of high altitude populations with low altitude populations of the same species found that the in high altitude environments, the ACE index of three species is significantly higher, the shannon index of dogs and macaques is significantly higher, and the simpson index of dogs is significantly lower (*p* < 0.05).

**Figure 3:**
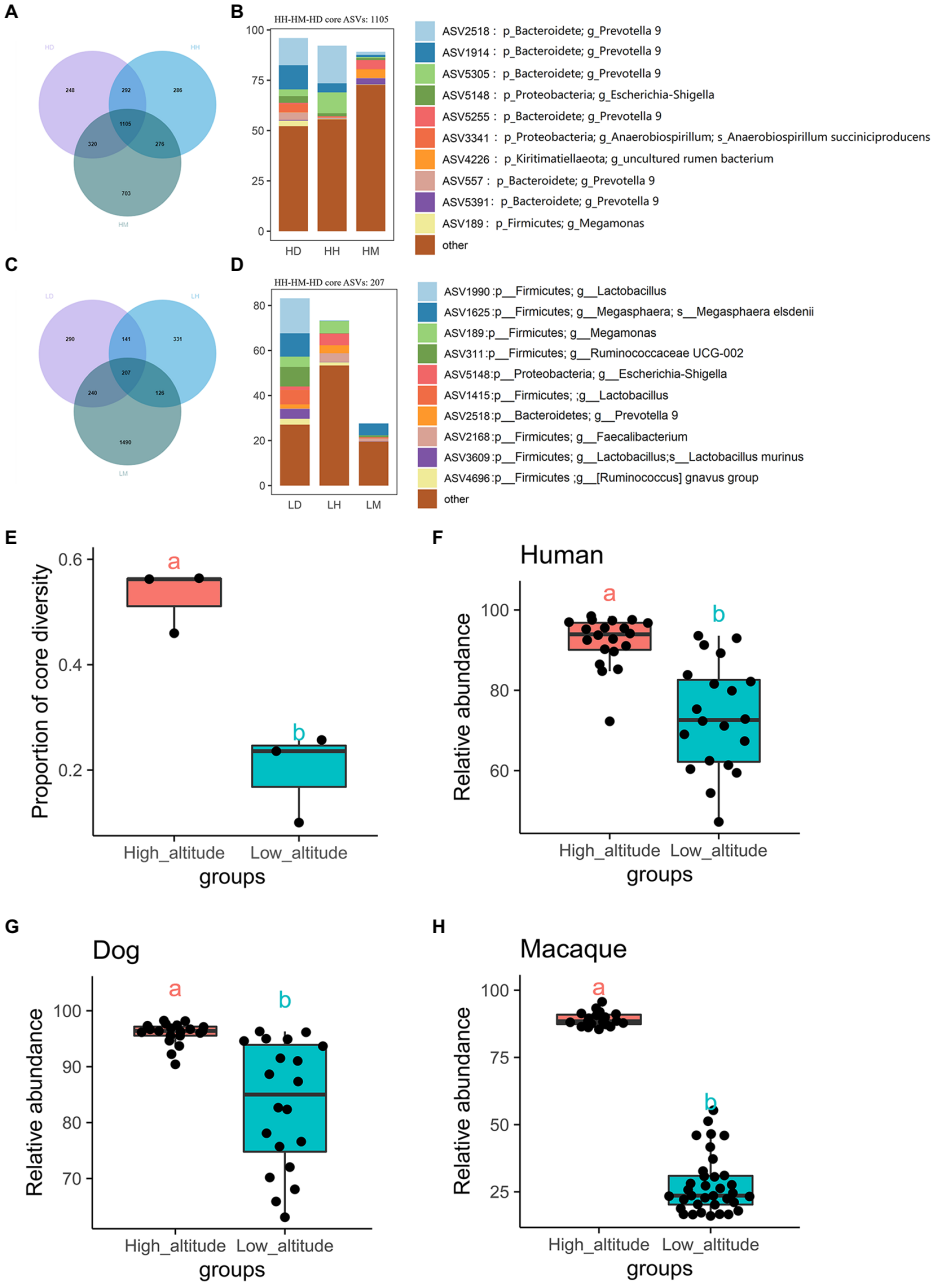
Venn diagram and percentage histogram of core ASV between different groups. **(A, C)** Numbers in plots are marked for how many ASVs are in this part. **(B, D)** The histogram represents the relative abundance of core ASVs. **(E)** Differential analysis of the number of core shared microbial species as a percentage of the total number of species in the high and low altitude populations(T-test). **(F–H)** Differential analysis of relative abundances of core shared microbiota between the three species at high and low altitude (T-test). The same letter indicates the difference is not significant (*p* > 0.05), HH stands for high altitude humans, HD stands for high altitude dogs, HM stands for high altitude wild macaques, LH stands for low altitude humans, LD stands for low altitude dogs, LM stands for low altitude wild macaques.

VN map analysis found 1,105 core shared ASVs (core microbiota) among wild macaques, humans, and dogs in high altitude environments, which accounted for 45.97%, 56.41%, and 56.23% of the proportion in the total types in each species, respectively (Figure 3A, Supplementary Table S2). The relative abundances of these core microbiota in wild macaques, humans, and dogs gut microbiota in high altitude environments were 89.16%, 92.18%, and 95.97%, respectively (Figure 3B; Supplementary Table S2). The relative abundance of the core microbiota between humans and dogs is more than 97%, however, there were 207 core shared ASVs among wild macaques, humans, and dogs at low altitudes, which accounted for 10.03%, 25.71%, and 23.58% of the proportion in the total types in each species, respectively (Figure 3C; Supplementary Table S2), and the relative abundances of these core microbiota in the gut microbiota of wild macaques, human, and dogs at low altitude were 27.65%, 73.39%, and 83.19%, respectively (Figure 3D; Supplementary Table S2). In these three subjects, the proportion and relative abundance of the core bacteria types in each subjects were significantly higher in high altitude environments than in low altitude environments (*p* < 0.05; Figures 3E–H), indicating that the gut microbiota composition of wild macaques and dogs was significantly more similar to that of humans at high altitude. At the same altitude, comparisons among different species also found that the number of core shared ASVs, the proportion and relative abundance of core bacterial types were higher in high altitude populations than in low altitude populations (Supplementary Table S3). In addition, it was found that the number of species-specific ASVs of wild macaques, humans, and dogs was also higher in high altitude environments than that at low altitude (HH: 1,281, HM: 1,229, HD: 1,409), but the abundance of these specific ASVs was lower, however, the relative abundance of core shared ASVs in high altitude environments of the same species was above 75%. The 578 core shared ASVs were identified in humans at high and low altitudes, and the relative abundance of these shared ASVs was 77.54% at high altitudes and 94.95% at low altitudes. There were 1,175 core shared ASVs in wild macaques at high and low altitudes, and the relative abundance of these core shared ASVs was 84.13% at high altitudes and 81.87% at low altitudes. There were 556 core shared ASVs in dogs at high and low altitudes, and the relative abundance of these core shared ASVs was 85.93% at high altitudes and 95.57% at low altitudes. This shows that the core microbiota in the same species is conservative (Supplementary Figure S1).

**Figure 4:**
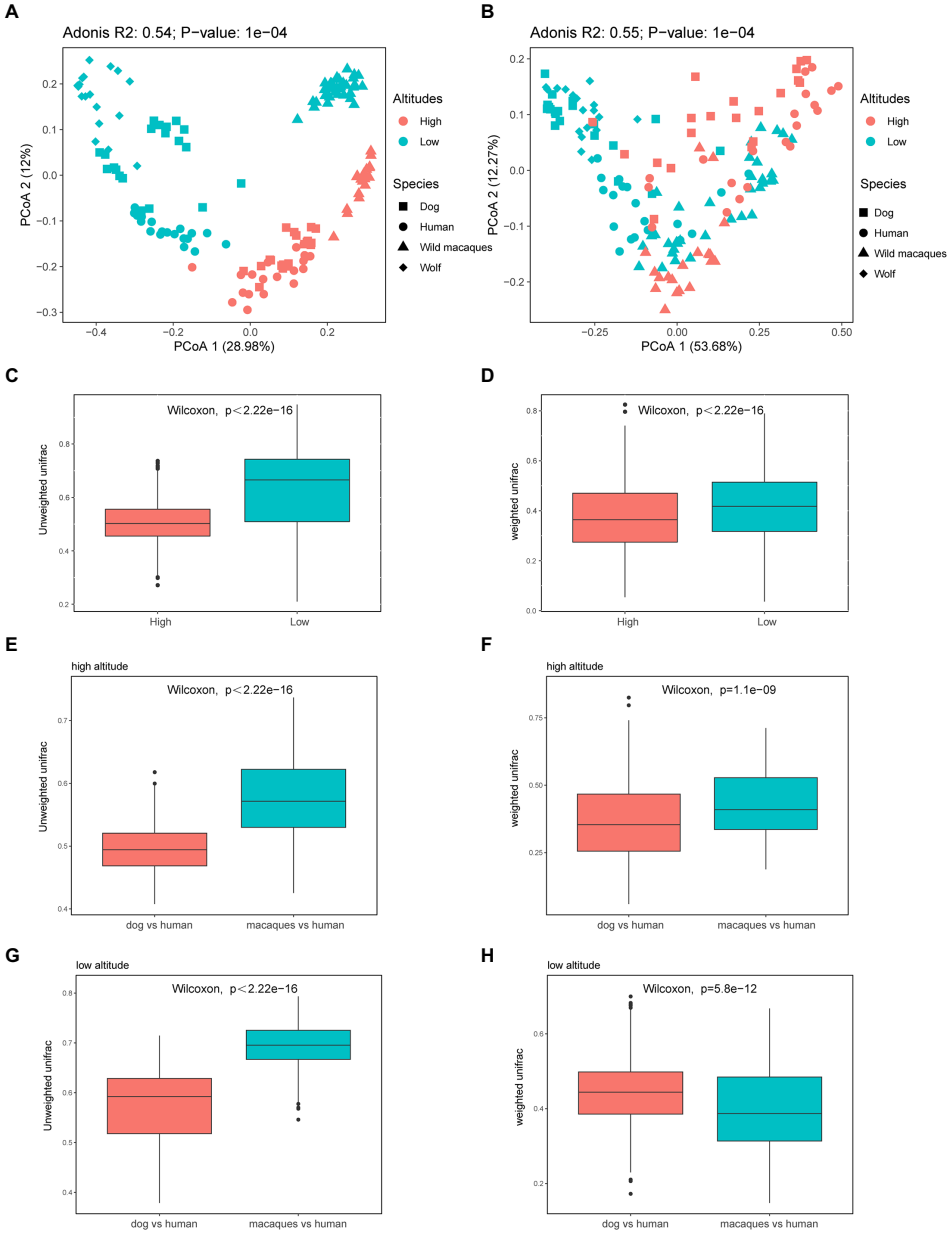
Microbiota beta diversity analysis of fecal samples among wild macaques, humans, and dogs in high altitude and low altitude environments. **(A)** PCoA plot using unweighted UniFrac distances dissimilarity based on ASVs in different groups. **(B)** PCoA plot using weighted UniFrac distances dissimilarity based on ASVs in different groups. **(C)** Similarity analysis between different species at high and low altitude based on unweighted UniFrac distances in wild macaques, humans, and dogs (Wilcoxon rank sum test). **(D)** Similarity analysis between different species at high and low altitude based on weighted UniFrac distances in wild macaques, humans, and dogs (Wilcoxon rank sum test). **(E,G)** Similarity analysis based on unweighted UniFrac distances in between humans and dogs and between humans and macaques (Wilcoxon rank sum test). **(F,H)** Similarity analysis based on weighted UniFrac distances in between humans and dogs and between humans and macaques (Wilcoxon rank sum test). The same letter indicates the difference is not significant (*p* > 0.05). HH stands for high altitude humans, HD stands for high altitude dogs, HM stands for high altitude wild macaques, LH stands for low altitude humans, LD stands for low altitude dogs, LM stands for low altitude wild macaques.

The distribution of beta diversity measures (weighted and unweighted UniFrac distances) was compared for the different geographical populations. PCoA was used to show the patterns of separation among different groups. PCoA analysis based on unweighted UniFrac distance shows that distinct clusters were clearly formed between the same species in high altitude and low altitude environments, and the distance between human and dog in the same altitude environments was significantly smaller than that between humans and macaques (Figure 4A). PCoA analysis based on weighted UniFrac distance shows that there is no obvious separation between different groups (Figure 4B). And the comparison between different species at the same altitude shows that the distance between dogs and wolves at low altitude is closer and that between humans and dogs at high altitude is closer (Figure 4B). The Wilcoxon rank sum test, based on weighted and unweighted UniFrac distance among different species at high and low altitudes, also found that the distance among wild macaques, humans, and dogs gut microbiota at high altitudes were significantly lower than that at low altitudes (*p* < 0.05, Figures 4C,D), which fully showed that the similarity of gut microbiota composition of wild macaques, humans, and dogs at high altitudes was significantly higher. Comparisons between different species also found that unweighted UniFrac distance between humans and dogs was significantly smaller than between humans and macaques in high altitude and low altitude environments (*p* < 0.05, Figures 4E,G). The weighted UniFrac distance between human and dog was significantly smaller than that between humans and macaques in high altitude environments (*p* < 0.05, Figure 4F), whereas the opposite was true in low altitude environments (*p* > 0.05, Figure 4H). In addition, our PCoA analysis of the gut microbiota compositional structures between dissimilar human sex and ages based on the weighted and unweighted UniFrac distances, in humans, the gut microbiota composition of individuals older than 30 years and younger than 30 years of age was not clearly separated, and adonis analysis also showed that there was no significant difference in gut microbiota composition between individuals older than 30 years and those younger than 30 years of age (*p* > 0.05, Supplementary Figures S2A,B). There were also no significant differences between the sexes (*p* > 0.05, Supplementary Figures S2C,D).

### 3.2. Taxonomy-based comparisons of gut microbiota

**Figure 5:**
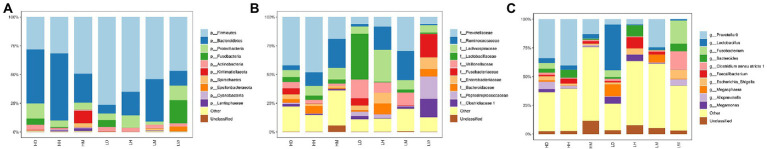
Gut microbiota taxonomic composition. Composition of gut microbiota among different groups at **(A)** phylum level, **(B)** family level and **(C)** genus level. The same letter indicates the difference is not significant (*p* > 0.05). HH stands for high altitude humans, HD stands for high altitude dogs, HM stands for high altitude wild macaques, LH stands for low altitude humans, LD stands for low altitude dogs, LM stands for low altitude wild macaques, LW stands for low Coyote.

Across all ASVs, the taxonomic analysis identified 29 known bacterial phyla, 184 families, and 478 genera. At the phylum level, the gut microbiota of wild macaques, humans, and dogs are dominated by Firmicutes, Bacteroidetes, Proteobacteria, Fusobacteria, and Actinobacteria (average relative abundance >1%, Figure 5A). At the family level, the predominant bacterial families isolated were Prevotellaceae, Ruminococcaceae, Lachnospiraceae, Lactobacillaceae, and Veillonellaceae (mean relative abundance >5%; Figure 5B). At the genus level, the predominant bacterial genera isolated were *Prevotella 9*, *Lactobacillus*, *Fusobacterium*, *Bacteroides*, *Clostridium sensu stricto 1*, and *Faecalibacterium* (mean relative abundance >3%; Figure 5C).

**Figure 6:**
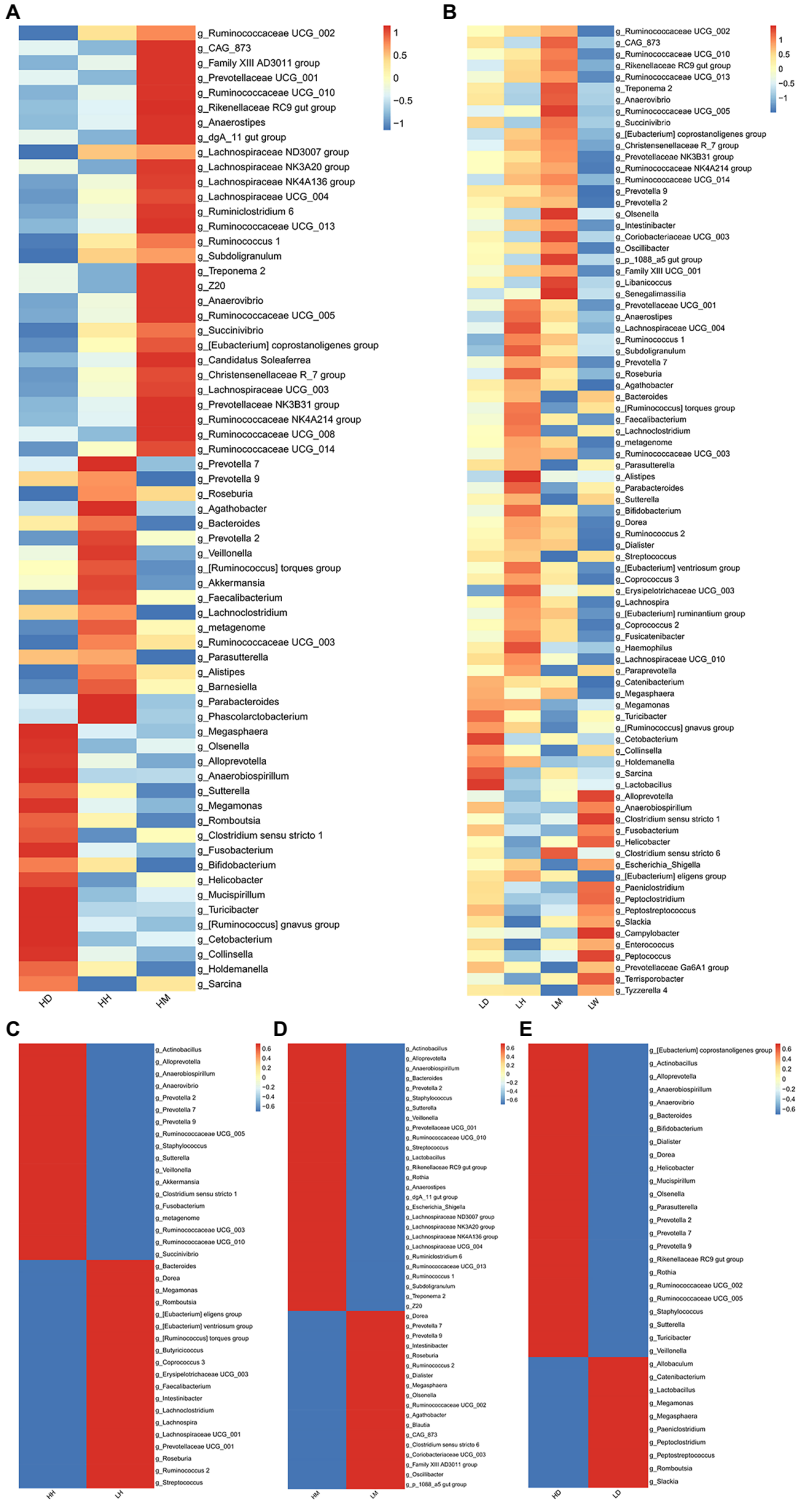
Heatmap showing the genus level LEFSe test (LDA > 2, *p* < 0.05) of gut microbiota among different species in the same altitude environments and between different altitude environments in the same species. **(A)** Comparison among three species at high altitude, **(B)** Comparison among three species at low altitude, **(C)** Comparison between high altitude and low altitude of humans, **(D)** Comparison between high altitude and low altitude of macaques, **(E)** Comparison between high altitude and low altitude of dogs. HH stands for high altitude humans, HD stands for high altitude dogs, HM stands for high altitude wild macaques, LH stands for low altitude humans, LD stands for low altitude dogs, LM stands for low altitude wild macaques, LW stands for low Coyote.

To further characterize the microbiota in the gut of different species, we performed LEfSe analysis (LDA > 2, *p* < 0.05) of the relative abundances at the genus level of the gut microbiota of different species in the high- and low altitude environments and found that the *Prevotella 7*, *Roseburia*, *Agathobacter*, *Bacteroides*, *Faecalibacterium*, *Lachnoclostridium*, *Metagenome*, *Ruminococcaceae UCG 003*, *Parasutterella*, *Alistipes*, *Parabacteroides*, and *[Ruminococcus] torques group* were significantly more abundant in the human gut microbiota than wild macaques and dogs, and were significantly high altitude indicative (Figures 6A,B). The abundance of *Ruminococcaceae UCG 002*, *Ruminococcaceae UCG 010*, *Rikenellaceae RC9 gut group*, *Ruminococcaceae UCG 013*, *Treponema 2*, *Anaerovibrio*, *Ruminococcaceae UCG 005*, *Succinivibrio*, *Christensenellaceae R 7 group*, *Prevotellaceae NK3B31 group*, *Ruminococcaceae NK4A214 group*, *Ruminococcaceae UCG 014*, *[Eubacterium] coprostanoligenes group,* and *CAG 873* in the gut microbiota of wild macaques was significantly higher than that of humans and dogs and had a significant species indicator effect (Figures 6A,B). The *Megasphaera*, *Megamonas*, *Turicibacter*, *Cetobacterium*, *Collinsella*, *Holdemanella*, *Sarcina*, and *[Ruminococcus] gnavus group* are significantly more abundant in the gut microbiota of dogs than those of humans and wild macaques and have a significant indicator effect (Figures 6A,B). In addition, LEFSe analysis of the relative abundance of gut microbiota composition of humans, wild macaques, and dogs in high altitude and low altitude environments showed that the abundance of *Actinobacillus*, *Alloprevotella*, *Anaerobiospirillum*, *Prevotella 2*, *Staphylococcus*, *Sutterella*, and *Veillonella* in high altitude environments was significantly higher than that in low altitude environments and had significant high altitude environments indicators (Figures 6C–E), however, in the low altitude environments, no indicator microbiota with significantly higher relative abundance was found in the three species.

### 3.3. Gut microbiota community assembly process measurement

**Figure 7:**
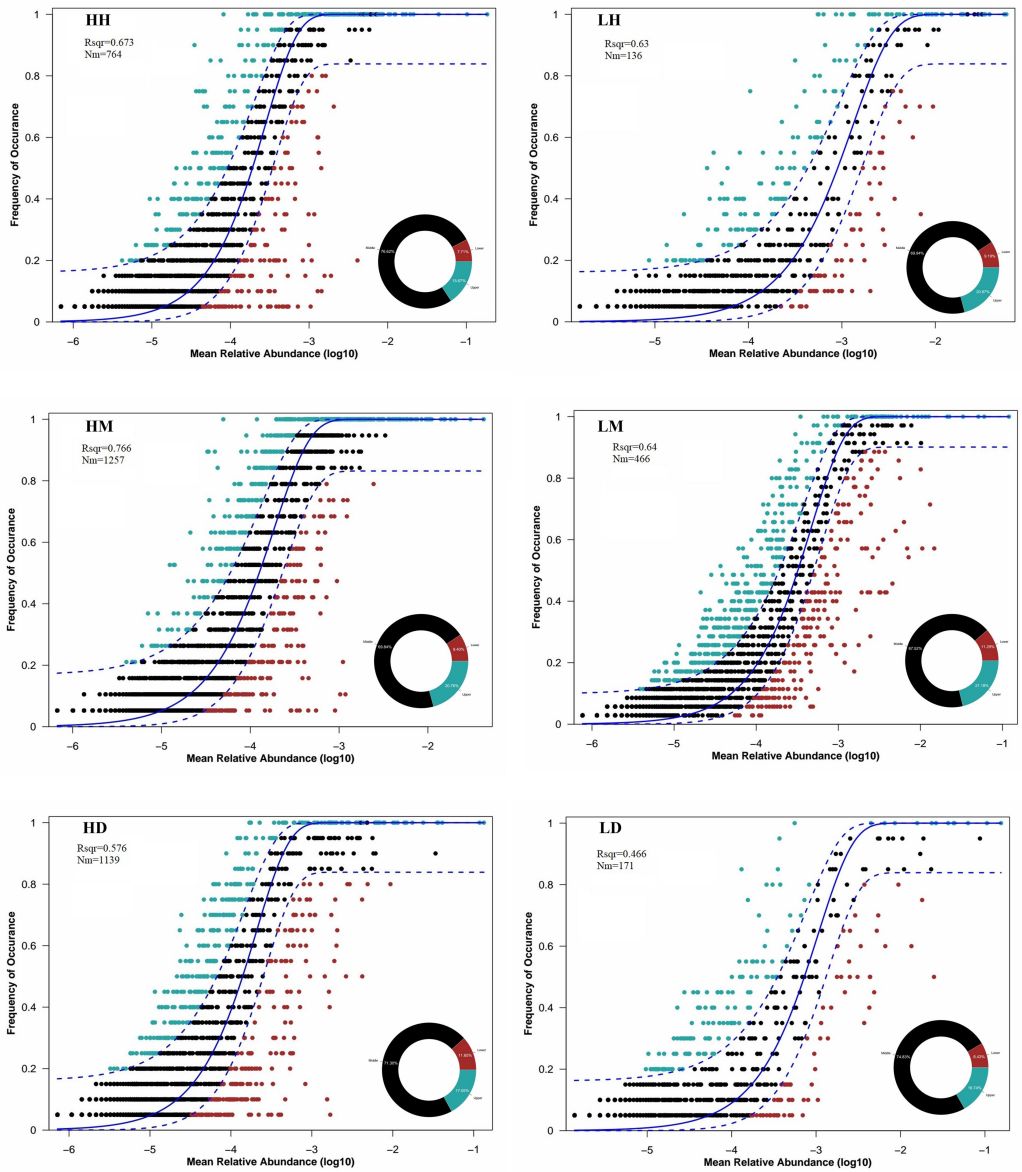
Quantitative results of the random process of gut microbiota community assembly in different groupings based on NCM. The solid black line represents the best fit to the NCM, and the dashed black line represents the 95% confidence interval around the model prediction. ASVs that occur more frequently or less frequently than NCM predictions are shown in different colors. “Nm” indicates the diffusion coefficient, and “Rsqr” indicates the fit to the model. HH stands for high altitude humans, HD stands for high altitude dogs, HM stands for high altitude wild macaques, LH stands for low altitude humans, LD stands for low altitude dogs, LM stands for low altitude wild macaques.

The analysis of their gut microbiota community assembly structure by NCM showed that the wild macaques, humans, and dogs gut microbiota in both high- and low altitude environments showed moderate fit to the neutral model (Figure 7). The goodness of fit of the models across species was in the following order for high- and low altitude populations: wild macaques (HM: *R*^2^ = 0.766, LM: *R*^2^ = 0.64) > humans (HH: *R*^2^ = 0.673, LH: *R*^2^ = 0.63) > dogs (HD: *R*^2^ = 0.576, LD: *R*^2^ = 0.466). Meanwhile, the fit of wild macaques, humans, and dogs gut microbiota in high altitude environments was higher than that in low altitude environments. This illustrates that the gut microbiota of wild macaques mostly influenced by stochastic processes, whether in high- or low altitude environments. The wild macaques, humans, and dogs gut microbiota communities in high altitude environments are all more influenced by stochastic processes than in low altitude environments. In addition, the product of metacommunity size and migration rate (Nm) value related to the gut microbiota community diffusion coefficient shows that wild macaques (HH: 764, HM: 1,257, HD: 1,139; LH: 136, LM: 466, LD: 171) are the diffusion coefficient largest in high altitude environments, followed by dogs and humans. Meanwhile, wild macaques, humans, and dogs in high altitude environments are higher diffusion coefficients of gut microbiota than those in low altitude environments. The migration rate ‘m’ of wild macaques, humans, and dogs in high altitude environments was significantly higher than that in low altitude environments (Figure 7).

## 4. Discussions

Host diet and phylogeny are two major factors that affect the composition and structure of gut microbiota (Groussin et al., 2017; Youngblut et al., 2019). In terms of phylogenetic relationship and morphology, the phylogenetic relationship between humans and wild macaques is closer, and the phylogenetic relationship between wolves and dogs is closer (dos Reis et al., 2012; Wang et al., 2013). Due to the early domestication of dogs by humans, the contact with humans is closer and the diet similarity is higher (Wang et al., 2013). In this study, the beta diversity analysis of different species at the same altitude found that the similarity between humans and dogs was greater than that between humans and wild macaques in gut microbiota compositional diversity, while low altitude dogs are more similar to wolves. Core shared microbiota analysis also found that the relative abundance of core shard microbiota was highest between humans and dogs, followed by between wolf and dogs, then finally between humans and wild macaques. Beta diversity analysis found that in high altitude environments, the weighted and unweighted UniFrac distance between humans and dogs is significantly smaller than that between humans and macaques (*p* < 0.05), and in low altitude environments, the unweighted UniFrac distance between humans and dogs is also significantly smaller than that between humans and macaques(*p* < 0.05). This is consistent with the results obtained by Coelho et al. (2018) through metagenomics studies in humans, dogs, mice, and pigs (Coelho et al., 2018). These results show that in the same altitude environments, similar diets promote the convergence of gut microbiota of dogs and humans. Meanwhile, our results also showed that the influence of genetic relationships on the composition of gut microbiota among different species seemed weak. This also corroborates findings in vertebrates that gut microbiota compositional differences between species are positively correlated with host evolutionary divergence times and that gut microbiota composition is more similar within host species than between species (Moeller et al., 2017; Song et al., 2020; Dillard et al., 2022). In addition, in this study, alpha diversity analysis found that the gut microbiota diversity of wild macaques with a more complex diet is much higher than that of humans and dogs with simpler diets (*p* < 0.05), which indicates that the gut microbiota composition of wild macaques has a higher diversity. During the sampling period, we observed that the diets of humans and dogs were similar, consisting mainly of rice, noodles, meat, fruits, and vegetables, however, wild macaques mainly eat leaves and fruits with a higher content of cellulose and lignin, which may provide additional resources to increase the diversity of the gut microbiota. Wild macaques living in wild natural environments (e.g., soil, larger scale, seasonality, social interactions) are also exposed to a more diverse microbial community compared to humans and dogs (Raulo et al., 2018; Trosvik et al., 2018). Limited by neutral dispersal, the more environmental microbial species the host is in contact with, the more likely the microbial species remain in the host (Burns et al., 2016; Clayton et al., 2018; Ross et al., 2018), therefore, the gut microbiota of wild macaques is more alpha diverse than that of humans and dogs.

The mammalian gut microbiota is shaped by the dispersal of organisms into habitats, followed by natural selection (i.e., habitat filtration), drift, and *in situ* diversification (Vellend, 2010). A comparison of mammalian phylogenies suggests that differences in selective pressures between the intestinal environments of mammalian species contribute to the diversification of the gut microbiota (Moeller et al., 2017). Geographic proximity and predator–prey interactions enable gut microbiota to flow between distantly related host species, resulting in the convergence of gut microbiota belonging to carnivorous and herbivorous mammals of different taxonomic purposes (Moeller et al., 2017). Studies have also found that drift and selection in the environment will also affect the assemblage of gut microbiota that inhabit animals (Trosvik et al., 2018), however, the low temperature, low oxygen, and high ultraviolet intensity in a high altitude environments pose a great challenge to the survival of animals (Simonson et al., 2010; Yu et al., 2016; Zhu et al., 2021). Previous studies have shown that in the long-term hypoxic environment, the Tibetan genotype changes toward environmental adaptability (Beall, 2011), while also driving changes in the composition structure of the gut microbiota. The more compositionally diverse gut microbiota is also able to promote the stability of the gut micro-ecosystem, increase the rate of dietary fermentation of the host, and help the host adapt to the high altitude environments (Li and Zhao, 2015; Zhang et al., 2016). Similar results were obtained in our study. The alpha diversity showed that the ACE index of humans, wild macaques, and dogs gut microbiota in high altitude environments was significantly higher than that in low altitude environments. However, there is no significant difference between the Simpson index of human and macaque in the high altitude environments and the low altitude environments, and there is no significant difference between the Shannon index of human in the high altitude environments and the low altitude environments. These results show that there are more species of gut microbiota in humans, wild macaques, and dogs at high altitude, but some unique microbiota in humans and wild macaques only exist in a few individuals. Beta diversity results showed that both weighted and unweighted UniFrac distances of wild macaques, humans, and dogs were significantly smaller in high altitude environments than in low altitude populations (*p* < 0.05), and the similarity of gut microbiota composition was significantly higher than that in low altitude environments. Core shared microbiota analysis also found that the species ratio and relative abundance of the core microbiota of each subject were significantly higher in high altitude environments than in low altitude environments (*p* < 0.05). These results strongly indicate that the convergence and sharing of gut microbiota among wild macaques, humans, and dogs are more significant in the high altitude environments, which strongly drives the convergence and adaptation of gut microbiota among wild macaques, humans, and dogs. In addition, beta diversity analysis found that the clustering of gut microbiota among the three species at high and low altitudes was more obvious in unweighted UniFrac distance than in weighted UniFrac distance. At high altitude, the weighted and unweighted UniFrac distance between humans and dogs was significantly lower than that between humans and macaques (*p* < 0.05). Core shared microbiota analysis also found that the relative abundance of human and dog core microbiota reached more than 89% in low altitude environments and 97% in high altitude environments. These results suggest that the contribution of altitude to the convergent adaptation of the gut microbiota of wild macaques, humans, and dogs to high altitude environments is mainly reflected in the compositional diversity of the microbiota, which indirectly affects the core shared microbiota abundance among different species. And at high altitude environments, similar diets promote their further convergent in terms of diversity and abundance.

NCM analysis also found that the extent of gut microbiota dispersal and influence by stochastic factors were higher in the high altitude wild macaque, human, and dog populations. This may be due to the fact that the lifestyle of human beings in the high altitude areas we sampled is mainly based on grazing and under forest collection, and the grazing area and under forest collection area are also the main habitats of wild macaques. The dog samples are from the captive populations of local residents, which have a high degree of niche overlap. As a result, the contact among species is closer than that of low altitude populations, and the selection pressure of low temperature, low oxygen, and high-intensity ultraviolet rays in a high altitude environments jointly leads to the diffusion, migration, and fusion of gut microbiota among different species. At high altitude, the stress of extreme environments, and natural resource dependent life patterns of humans (grazing and understory harvesting), are closely related and are also important factors driving the convergence of gut microbiota from wild macaques, humans, and dogs at high altitude.

Taxonomic composition analysis found that Firmicutes and Bacteroidetes were dominant among the three mammalians’ gut microbiota, which was consistent with previous findings (Duncan et al., 2008; Fogel, 2015). The LEFSe analysis showed that the abundance of *Actinobacillus*, *Alloprevotella*, *Anaerobiospirillum*, *Prevotella 2*, *Staphylococcus*, *Sutterella*, and *Veillonella* in wild macaques, humans, and dogs in the high altitude environments is significantly higher than that in the low altitude environments and has a significant role in indicating high altitude environments, however, in the low altitude environments, no indicator microbiota with a significantly higher relative abundance was found in the three species. Previous studies have found that *Actinobacillus* is significantly positively correlated with systolic blood pressure in blood pressure regulation (Chen B. Y. et al., 2022). *Alloprevotella* can utilize carbohydrates and undergo fermentation to produce acetate and succinate, two major end metabolites (Xiao et al., 2013), while also having a cardiovascular risk-reducing effect (Kong et al., 2019). However, altitude has a positive linear relationship with systolic blood pressure (SBP) and an important effect on host blood pressure (Aryal et al., 2016). These results indicates that the high abundance of *Actinobacillus* and *Alloprevotella* can help the host regulate blood pressure and adapt to the high altitude hypoxic environment. *Anaerobiospirillum* is isolated from the feces of dogs and cats. It can use glucose metabolism to produce succinic and acetic acids but may also form trace lactic and formic acids (Davis et al., 1976). *Prevotella* is a probiotic widely distributed in the gut of animals, which helps to decompose protein and carbohydrates (Davis et al., 1976; Kovatcheva-Datchary et al., 2015). *Sutterella* was confirmed to be associated with obesity in mice (Liu et al., 2018). This indicates that these microbiotas were significantly more abundant in the gut of wild macaques, humans, and dogs at high altitudes, which will promote the host to digest and decompose food and produce energy substances, and help the host to adapt to the high energy demand in the high altitude environments. *Veillonella* is a kind of microbiota that can enhance performance, using lactic acid as a carbon source, it can quickly decompose lactic acid into propionic acid, thereby reducing the concentration of lactic acid and improving sports performance (Scheiman et al., 2019). The *Veillonella* in high abundance is able to improve host tolerance, prompting its adaptation to high altitude environments. *Staphylococcus*, were found in the Berry, typically causes surgical and skin infections, respiratory diseases, and food poisoning (Licitra, 2013), however, the reasoning as to why the abundance of *Staphylococcus* in the intestines of wild macaques, humans, and dogs at high altitudes is significantly higher than that at low altitudes needs to be revealed. These results indicate that these common characteristic bacteria play an important role in the adaptation of wild macaques, humans, and dogs to high altitude environments such as energy compensation and hypoxia adaptation.

In conclusion, our results show that diet and high altitude strongly drive convergent adaptation of gut microbiota in wild macaques, humans, and dogs to high altitude environments. Among them, the contribution of high altitude environments to the convergent adaptation of the gut microbiota of wild macaques, humans, and dogs are mainly reflected in the compositional diversity of the microbiota, which indirectly affects the core shared microbiota abundance among different species. And at high altitude environments, similar diets promote their further convergent in terms of diversity and abundance. Meanwhile, the convergence of intestinal microbiota in animals at high altitudes may be partly due to microbial diffusion between hosts. In addition, the microbiota is significantly enriched in wild macaques, humans, and dogs from high altitude environments and plays an important role in the hosts energy compensation and cardiovascular regulation and helping the host adapt to the high energy demand and low oxygen pressure of high altitude environments.

## Data availability statement

The datasets presented in this study can be found in online repositories. The names of the repository/repositories and accession number(s) can be found below: https://www.ncbi.nlm.nih.gov/, PRJNA760678.

## Ethics statement

Before sample collection, all the human work was approved by the Animal Welfare and Animal Ethics Committee of Sichuan Agricultural University (SKY-S20171006). The patients/participants provided their written informed consent to participate in this study. Before sample collection, all the animal work was approved by the Animal Welfare and Animal Ethics Committee of Sichuan Agricultural University (SKY-S20171006). Written informed consent was obtained from the owners for the participation of their animals in this study.

## Author contributions

HuX, JZ, and YY designed the experiment and wrote the first draft. MD, HoX, SY, YX, DL, MX, QN, and MZ collected the fecal samples and performed preliminary preparation. All authors have helped in revision and approved the final manuscript.

## Funding

This work was supported by the National Natural Science Foundation of China under Grant (31870355).

## Conflict of interest

The authors declare that the research was conducted in the absence of any commercial or financial relationships that could be construed as a potential conflict of interest.

## Publisher’s note

All claims expressed in this article are solely those of the authors and do not necessarily represent those of their affiliated organizations, or those of the publisher, the editors and the reviewers. Any product that may be evaluated in this article, or claim that may be made by its manufacturer, is not guaranteed or endorsed by the publisher.

## Abbreviations

ASV: Amplicon sequence variants
VFAs: volatile fatty acids
VN: Venn
LEfSe: Linear Discriminant Analysis (LDA) Effect Size
ANOSIM: Analysis of similarities
PCoA: Principal Co-ordinates Analysis
NCM: neutral community model
FMT: Fecal microbial transmission
GF: Germ-free
SBP: Systolic blood pressure

## References

[ref1] Aryal N. Weatherall M. Bhatta Y. K. Mann S. (2016). Blood pressure and hypertension in adults permanently living at high altitude: a systematic review and meta-analysis. High Alt. Med. Biol. 17, 185–193. doi: 10.1089/ham.2015.0118, PMID: 27575245

[ref2] Bäckhed F. Ley R. E. Sonnenburg J. L. Peterson D. A. Gordon J. I. (2005). Host-bacterial mutualism in the human intestine. Science 307, 1915–1920. doi: 10.1126/science.1104816, PMID: 15790844

[ref3] Beall C. M. (2011). Genetic changes in Tibet. High Alt. Med. Biol. 12, 101–102. doi: 10.1089/ham.2011.1007 21718152

[ref4] Blaut M. Collins M. D. Welling G. W. Doré J. Van Loo J. De Vos W. (2002). Molecular biological methods for studying the gut microbiota: the EU human gut flora project. Br. J. Nutr. 87, 203–211. doi: 10.1079/BJNBJN/2002539, PMID: 12088520

[ref5] Burns A. R. Stephens W. Z. Stagaman K. Wong S. Rawls J. F. Guillemin K.. (2016). Contribution of neutral processes to the assembly of gut microbial communities in the zebrafish over host development. ISME J. 10, 655–664. doi: 10.1038/ismej.2015.142, PMID: 26296066 PMC4817674

[ref6] Callahan B. J. Mcmurdie P. J. Rosen M. J. Han A. W. Johnson A. J. Holmes S. P. (2016). DADA2: high-resolution sample inference from Illumina amplicon data. Nat. Methods 13, 581–583. doi: 10.1038/nmeth.3869, PMID: 27214047 PMC4927377

[ref7] Chan A. W. Chong K. Y. Martinovich C. Simerly C. Schatten G. (2001). Transgenic monkeys produced by retroviral gene transfer into mature oocytes. Science 291, 309–312. doi: 10.1126/science.291.5502.309, PMID: 11209082

[ref8] Chen B.-Y. Lin W.-Z. Li Y.-L. Bi C. Du L.-J. Liu Y.. (2022). Roles of oral microbiota and oral-gut microbial transmission in hypertension. J. Adv. Res. 43, 147–161. doi: 10.1016/j.jare.2022.03.007, PMID: 36585105 PMC9811375

[ref9] Chen T. Liu Y.-X. Huang L. (2022). ImageGP: an easy-to-use data visualization web server for scientific researchers. iMeta 1:e5. doi: 10.1002/imt2.5 PMC10989750 38867732

[ref10] Chen T. Zhang H. Liu Y. Liu Y. X. Huang L. (2021). EVenn: easy to create repeatable and editable Venn diagrams and Venn networks online. J. Genet. Genomics 48, 863–866. doi: 10.1016/j.jgg.2021.07.007, PMID: 34452851

[ref11] Chen W. Ren K. Isabwe A. Chen H. Liu M. Yang J. (2019). Stochastic processes shape microeukaryotic community assembly in a subtropical river across wet and dry seasons. Microbiome 7:138. doi: 10.1186/s40168-019-0749-8, PMID: 31640783 PMC6806580

[ref12] Clayton J. B. Gomez A. Amato K. Knights D. Travis D. A. Blekhman R.. (2018). The gut microbiome of nonhuman primates: lessons in ecology and evolution. Am. J. Primatol. 80:e22867. doi: 10.1002/ajp.22867, PMID: 29862519

[ref13] Coelho L. P. Kultima J. R. Costea P. I. Fournier C. Pan Y. Czarnecki-Maulden G.. (2018). Similarity of the dog and human gut microbiomes in gene content and response to diet. Microbiome 6:72. doi: 10.1186/s40168-018-0450-3, PMID: 29669589 PMC5907387

[ref14] Davis C. P. Cleven D. Brown J. L. Balish E. J. I. J. O. S. Microbiology E. (1976). Anaerobiospirillum, a new genus of spiral-shaped bacteria. Int. J. Syst. Evol. Microbiol. 26, 498–504. doi: 10.1099/00207713-26-4-498

[ref15] De Filippo C. Cavalieri D. Di Paola M. Ramazzotti M. Poullet J. B. Massart S.. (2010). Impact of diet in shaping gut microbiota revealed by a comparative study in children from Europe and rural Africa. Proc. Natl. Acad. Sci. U. S. A. 107, 14691–14696. doi: 10.1073/pnas.1005963107, PMID: 20679230 PMC2930426

[ref16] Dillard B. A. Chung A. K. Gunderson A. R. Campbell-Staton S. C. Moeller A. H. (2022). Humanization of wildlife gut microbiota in urban environments. elife 11:e76381. doi: 10.7554/eLife.76381 35638605 PMC9203057

[ref17] Dominguez-Bello M. G. Costello E. K. Contreras M. Magris M. Hidalgo G. Fierer N.. (2010). Delivery mode shapes the acquisition and structure of the initial microbiota across multiple body habitats in newborns. Proc. Natl. Acad. Sci. U. S. A. 107, 11971–11975. doi: 10.1073/pnas.1002601107, PMID: 20566857 PMC2900693

[ref18] Dos Reis M. Inoue J. Hasegawa M. Asher R. J. Donoghue P. C. Yang Z. (2012). Phylogenomic datasets provide both precision and accuracy in estimating the timescale of placental mammal phylogeny. Proc. Biol. Sci. 279, 3491–3500. doi: 10.1098/rspb.2012.0683, PMID: 22628470 PMC3396900

[ref19] Duncan S. H. Lobley G. E. Holtrop G. Ince J. Johnstone A. M. Louis P.. (2008). Human colonic microbiota associated with diet, obesity and weight loss. Int. J. Obes. 32, 1720–1724. doi: 10.1038/ijo.2008.155, PMID: 18779823

[ref20] Fackelmann G. Gillingham M. A. F. Schmid J. Heni A. C. Wilhelm K. Schwensow N.. (2021). Human encroachment into wildlife gut microbiomes. Commun. Biol. 4:800. doi: 10.1038/s42003-021-02315-7, PMID: 34172822 PMC8233340

[ref21] Fadrosh D. W. Ma B. Gajer P. Sengamalay N. Ott S. Brotman R. M.. (2014). An improved dual-indexing approach for multiplexed 16S rRNA gene sequencing on the Illumina MiSeq platform. Microbiome 2:6. doi: 10.1186/2049-2618-2-6, PMID: 24558975 PMC3940169

[ref22] Fogel A. T. (2015). The gut microbiome of wild lemurs: a comparison of sympatric Lemur catta and Propithecus verreauxi. Folia Primatol (Basel). 86, 85–95. doi: 10.1159/000369971, PMID: 26022304

[ref23] Goodrich J. K. Waters J. L. Poole A. C. Sutter J. L. Koren O. Blekhman R.. (2014). Human genetics shape the gut microbiome. Cells 159, 789–799. doi: 10.1016/j.cell.2014.09.053, PMID: 25417156 PMC4255478

[ref24] Groussin M. Mazel F. Sanders J. G. Smillie C. S. Lavergne S. Thuiller W.. (2017). Unraveling the processes shaping mammalian gut microbiomes over evolutionary time. Nat. Commun. 8:14319. doi: 10.1038/ncomms14319, PMID: 28230052 PMC5331214

[ref25] Hall M. Beiko R. G. (2018). 16S rRNA gene analysis with QIIME2. Methods Mol. Biol. 1849, 113–129. doi: 10.1007/978-1-4939-8728-3_8 30298251

[ref26] Huang G. Wang L. Li J. Hou R. Wang M. Wang Z.. (2022). Seasonal shift of the gut microbiome synchronizes host peripheral circadian rhythm for physiological adaptation to a low-fat diet in the giant panda. Cell Rep. 38:110203. doi: 10.1016/j.celrep.2021.110203, PMID: 35045306

[ref27] Huang G. Wang X. Hu Y. Wu Q. Nie Y. Dong J.. (2021). Diet drives convergent evolution of gut microbiomes in bamboo-eating species. Sci. China Life Sci. 64, 88–95. doi: 10.1007/s11427-020-1750-7, PMID: 32617829

[ref28] Kong C. Gao R. Yan X. Huang L. Qin H. (2019). Probiotics improve gut microbiota dysbiosis in obese mice fed a high-fat or high-sucrose diet. Nutrition 60, 175–184. doi: 10.1016/j.nut.2018.10.002 30611080

[ref29] Kovatcheva-Datchary P. Nilsson A. Akrami R. Lee Y. S. De Vadder F. Arora T.. (2015). Dietary fiber-induced improvement in glucose metabolism is associated with increased abundance of Prevotella. Cell Metab. 22, 971–982. doi: 10.1016/j.cmet.2015.10.001, PMID: 26552345

[ref30] Ley R. E. Hamady M. Lozupone C. Turnbaugh P. J. Ramey R. R. Bircher J. S.. (2008). Evolution of mammals and their gut microbes. Science 320, 1647–1651. doi: 10.1126/science.1155725, PMID: 18497261 PMC2649005

[ref31] Ley R. E. Peterson D. A. Gordon J. I. (2006). Ecological and evolutionary forces shaping microbial diversity in the human intestine. Cells 124, 837–848. doi: 10.1016/j.cell.2006.02.017, PMID: 16497592

[ref32] Li H. Li T. Beasley D. E. Heděnec P. Xiao Z. Zhang S.. (2016). Diet diversity is associated with Beta but not alpha diversity of Pika gut microbiota. Front. Microbiol. 7:1169. doi: 10.3389/fmicb.2016.01169, PMID: 27512391 PMC4961685

[ref33] Li K. Dan Z. Gesang L. Wang H. Zhou Y. Du Y.. (2016). Comparative analysis of gut microbiota of native Tibetan and Han populations living at different altitudes. PLoS One 11:e0155863. doi: 10.1371/journal.pone.0155863, PMID: 27232599 PMC4883765

[ref34] Li L. Zhao X. (2015). Comparative analyses of fecal microbiota in Tibetan and Chinese Han living at low or high altitude by barcoded 454 pyrosequencing. Sci. Rep. 5:14682. doi: 10.1038/srep14682, PMID: 26443005 PMC4595765

[ref35] Licitra G. (2013). Etymologia: staphylococcus. Emerg. Infect. Dis. 19:1553. doi: 10.3201/eid1909.ET1909

[ref36] Liu G. Bei J. Liang L. Yu G. Li L. Li Q. (2018). Stachyose improves inflammation through modulating gut microbiota of high-fat diet/Streptozotocin-induced type 2 diabetes in rats. Mol. Nutr. Food Res. 62:e1700954. doi: 10.1002/mnfr.201700954, PMID: 29341443

[ref37] Ma Y. Ma S. Chang L. Wang H. Ga Q. Ma L.. (2019). Gut microbiota adaptation to high altitude in indigenous animals. Biochem. Biophys. Res. Commun. 516, 120–126. doi: 10.1016/j.bbrc.2019.05.085, PMID: 31196622

[ref38] Moeller A. H. Foerster S. Wilson M. L. Pusey A. E. Hahn B. H. Ochman H. (2016). Social behavior shapes the chimpanzee pan-microbiome. Sci. Adv. 2:e1500997. doi: 10.1126/sciadv.1500997, PMID: 26824072 PMC4730854

[ref39] Moeller A. H. Suzuki T. A. Lin D. Lacey E. A. Wasser S. K. Nachman M. W. (2017). Dispersal limitation promotes the diversification of the mammalian gut microbiota. Proc. Natl. Acad. Sci. U. S. A. 114, 13768–13773. doi: 10.1073/pnas.1700122114, PMID: 29229828 PMC5748161

[ref40] Raulo A. Ruokolainen L. Lane A. Amato K. Knight R. Leigh S.. (2018). Social behaviour and gut microbiota in red-bellied lemurs (Eulemur rubriventer): in search of the role of immunity in the evolution of sociality. J. Anim. Ecol. 87, 388–399. doi: 10.1111/1365-2656.12781, PMID: 29205327

[ref41] Ross A. A. Müller K. M. Weese J. S. Neufeld J. D. (2018). Comprehensive skin microbiome analysis reveals the uniqueness of human skin and evidence for phylosymbiosis within the class Mammalia. Proc. Natl. Acad. Sci. U. S. A. 115, E5786–e5795. doi: 10.1073/pnas.1801302115, PMID: 29871947 PMC6016819

[ref42] Scheiman J. Luber J. M. Chavkin T. A. Macdonald T. Tung A. Pham L. D.. (2019). Meta-omics analysis of elite athletes identifies a performance-enhancing microbe that functions via lactate metabolism. Nat. Med. 25, 1104–1109. doi: 10.1038/s41591-019-0485-4, PMID: 31235964 PMC7368972

[ref43] Simonson T. S. Yang Y. Huff C. D. Yun H. Qin G. Witherspoon D. J.. (2010). Genetic evidence for high-altitude adaptation in Tibet. Science 329, 72–75. doi: 10.1126/science.1189406 20466884

[ref44] Song S. J. Sanders J. G. Delsuc F. Metcalf J. Amato K. Taylor M. W.. (2020). Comparative analyses of vertebrate gut microbiomes reveal convergence between birds and bats. MBio 11:e02901-19. doi: 10.1128/mBio.02901-19, PMID: 31911491 PMC6946802

[ref45] Sugden S. Sanderson D. Ford K. Stein L. Y. St Clair C. C. (2020). An altered microbiome in urban coyotes mediates relationships between anthropogenic diet and poor health. Sci. Rep. 10:22207. doi: 10.1038/s41598-020-78891-1, PMID: 33335116 PMC7746695

[ref46] Sugden S. St Clair C. C. Stein L. Y. (2021). Individual and site-specific variation in a biogeographical profile of the coyote gastrointestinal microbiota. Microb. Ecol. 81, 240–252. doi: 10.1007/s00248-020-01547-0, PMID: 32594248

[ref47] Trosvik P. De Muinck E. J. Rueness E. K. Fashing P. J. Beierschmitt E. C. Callingham K. R.. (2018). Multilevel social structure and diet shape the gut microbiota of the gelada monkey, the only grazing primate. Microbiome 6:84. doi: 10.1186/s40168-018-0468-6, PMID: 29729671 PMC5935910

[ref48] Tung J. Barreiro L. B. Burns M. B. Grenier J. C. Lynch J. Grieneisen L. E.. (2015). Social networks predict gut microbiome composition in wild baboons. elife 4:e05224. doi: 10.7554/eLife.05224, PMID: 25774601 PMC4379495

[ref49] Vaishampayan P. A. Kuehl J. V. Froula J. L. Morgan J. L. Ochman H. Francino M. P. (2010). Comparative metagenomics and population dynamics of the gut microbiota in mother and infant. Genome Biol. Evol. 2, 53–66. doi: 10.1093/gbe/evp057, PMID: 20333224 PMC2839348

[ref50] Vellend M. (2010). Conceptual synthesis in community ecology. Q. Rev. Biol. 85, 183–206. doi: 10.1086/652373 20565040

[ref51] Wang G. D. Zhai W. Yang H. C. Fan R. X. Cao X. Zhong L.. (2013). The genomics of selection in dogs and the parallel evolution between dogs and humans. Nat. Commun. 4:1860. doi: 10.1038/ncomms2814, PMID: 23673645

[ref52] Wang G. D. Zhai W. Yang H. C. Wang L. Zhong L. Liu Y. H.. (2016). Out of southern East Asia: the natural history of domestic dogs across the world. Cell Res. 26, 21–33. doi: 10.1038/cr.2015.147, PMID: 26667385 PMC4816135

[ref53] Wang X. Wu X. Shang Y. Gao Y. Li Y. Wei Q.. (2022). High-altitude drives the convergent evolution of alpha diversity and indicator microbiota in the gut microbiomes of ungulates. Front. Microbiol. 13:953234. doi: 10.3389/fmicb.2022.953234, PMID: 35875556 PMC9301279

[ref54] Wu Y. Yao Y. Dong M. Xia T. Li D. Xie M.. (2020). Characterisation of the gut microbial community of rhesus macaques in high-altitude environments. BMC Microbiol. 20:68. doi: 10.1186/s12866-020-01747-1, PMID: 32216756 PMC7098161

[ref55] Xia T. Yao Y. Wang C. Dong M. Wu Y. Li D.. (2021). Seasonal dynamics of gut microbiota in a cohort of wild Tibetan macaques (Macaca thibetana) in western China. Glob. Ecol. Conserv. 25:e01409. doi: 10.1016/j.gecco.2020.e01409

[ref56] Xiao X. Li Y. Xiao L. (2013). The novel species and genus discovered and nominated from the human oral cavity in 2009--2012. West China J. Stomatol. 31, 217–220. doi: 10.7518/hxkq.2013.02.026 23662572

[ref57] Xue Z. Zhang W. Wang L. Hou R. Zhang M. Fei L.. (2015). The bamboo-eating giant panda harbors a carnivore-like gut microbiota, with excessive seasonal variations. MBio 6, e00022–e00015. doi: 10.1128/mBio.00022-15, PMID: 25991678 PMC4442137

[ref58] Yan M. A. Xin X. Jiakai F. A. N. Benyin Z. (2021). Effect of altitude on the diversity of gut microbiota of yaks grazing on the Qinghai-Tibet plateau. Microbiol. China 49, 620–634. doi: 10.13344/j.microbiol.china.210752

[ref59] Yao R. Dai Q. Wu T. Yang Z. Chen H. Liu G.. (2021). Fly-over phylogeny across invertebrate to vertebrate: the giant panda and insects share a highly similar gut microbiota. Comput. Struct. Biotechnol. J. 19, 4676–4683. doi: 10.1016/j.csbj.2021.08.025, PMID: 34504662 PMC8390952

[ref60] Youngblut N. D. Reischer G. H. Walters W. Schuster N. Walzer C. Stalder G.. (2019). Host diet and evolutionary history explain different aspects of gut microbiome diversity among vertebrate clades. Nat. Commun. 10:2200. doi: 10.1038/s41467-019-10191-3, PMID: 31097702 PMC6522487

[ref61] Yu L. Wang G. D. Ruan J. Chen Y. B. Yang C. P. Cao X.. (2016). Genomic analysis of snub-nosed monkeys (Rhinopithecus) identifies genes and processes related to high-altitude adaptation. Nat. Genet. 48, 947–952. doi: 10.1038/ng.3615, PMID: 27399969

[ref62] Zeng B. Zhang S. Xu H. Kong F. Yu X. Wang P.. (2020). Gut microbiota of Tibetans and Tibetan pigs varies between high and low altitude environments. Microbiol. Res. 235:126447. doi: 10.1016/j.micres.2020.126447, PMID: 32114362

[ref63] Zhang W. Liu W. Hou R. Zhang L. Schmitz-Esser S. Sun H.. (2018). Age-associated microbiome shows the giant panda lives on hemicelluloses, not on cellulose. ISME J. 12, 1319–1328. doi: 10.1038/s41396-018-0051-y, PMID: 29391488 PMC5931968

[ref64] Zhang W. Xie J. Xia S. Fan X. Schmitz-Esser S. Zeng B.. (2022). Evaluating a potential model to analyze the function of the gut microbiota of the giant panda. Front. Microbiol. 13:1086058. doi: 10.3389/fmicb.2022.1086058, PMID: 36605506 PMC9808404

[ref65] Zhang Z. Xu D. Wang L. Hao J. Wang J. Zhou X.. (2016). Convergent evolution of rumen microbiomes in high-altitude mammals. Curr. Biol. 26, 1873–1879. doi: 10.1016/j.cub.2016.05.012, PMID: 27321997

[ref66] Zhao J. Yao Y. Li D. Xu H. Wu J. Wen A.. (2018). Characterization of the gut microbiota in six geographical populations of Chinese rhesus macaques (*Macaca mulatta*), implying an adaptation to high-altitude environment. Microb. Ecol. 76, 565–577. doi: 10.1007/s00248-018-1146-8, PMID: 29372281

[ref67] Zhu H. Zhong L. Li J. Wang S. Qu J. (2021). Differential expression of metabolism-related genes in plateau Pika (Ochotona curzoniae) at different altitudes on the Qinghai-Tibet plateau. Front. Genet. 12:784811. doi: 10.3389/fgene.2021.784811, PMID: 35126457 PMC8811202

