# Supplementary material for: Diet and high altitude strongly drive convergent adaptation of gut microbiota in wild macaques, humans, and dogs to high altitude environments
Source: Front Microbiol. 2023 Feb 23;14:1067240. doi: 10.3389/fmicb.2023.1067240 (PMC9995840; doi:10.3389/fmicb.2023.1067240)
Supplement: Supplementary file 1 [file Data_Sheet_1.PDF]

Table S1 Alpha diversity statistics significance test

ID	ACE index		Shannon_index		simpson_index	
	diff	p.adj	diff	p.adj	diff	p.adj
LD-LW	116.58889	4.95E-05	0.0587005	0.9999059	0.0283897	0.7522748
LH-LW	155.23889	2.10E-08	0.7815739	0.0006092	0.0492172	0.1406748
LM-LW	457.0746	3.55E-15	1.6381671	6.64E-14	0.0756997	0.0003491
HH-LW	478.08889	3.55E-15	1.0626398	6.63E-07	0.0389851	0.3933214
HD-LW	500.03889	3.55E-15	1.0959777	2.70E-07	0.0509304	0.1142599
HM-LW	867.49415	3.55E-15	2.5672496	3.55E-15	0.1175765	2.09E-07
LH-LD	38.65	0.635845	0.7228734	0.0013835	0.0776069	0.0009864
LM-LD	340.48571	3.55E-15	1.5794666	6.66E-14	0.1040894	6.40E-08
HH-LD	361.5	3.55E-15	1.0039393	1.51E-06	0.0673748	0.0071532
HD-LD	383.45	3.55E-15	1.0372772	6.11E-07	0.0793201	0.0006914
HM-LD	750.90526	3.55E-15	2.508549	3.55E-15	0.1459662	3.05E-11
LM-LH	301.83571	3.55E-15	0.8565932	4.11E-06	0.0264825	0.6777499
HH-LH	322.85	3.55E-15	0.2810659	0.6896801	0.0102321	0.9979746
HD-LH	344.8	3.55E-15	0.3144038	0.5655007	0.0017132	0.9999999
HM-LH	712.25526	3.55E-15	1.7856757	6.66E-14	0.0683593	0.0070217
HH-LM	21.014286	0.9473729	0.5755273	0.0061387	0.0367146	0.2868996
HD-LM	42.964286	0.3602755	0.5421894	0.0124069	0.0247693	0.742356
HM-LM	410.41955	3.55E-15	0.9290824	7.25E-07	0.0418768	0.166883
HD-HH	21.95	0.96362	0.0333378	0.9999961	0.0119453	0.9952265
HM-HH	389.40526	3.55E-15	1.5046097	8.76E-13	0.0785914	0.0009924
HM-HD	367.45526	3.55E-15	1.4712719	2.40E-12	0.0666461	0.0095061

Table S2 Diversity and relative abundance of core microbiota of humans, wild macaques and domestic dogs at different altitudes

Altitude	Groups	Number of ASVs	Number of cores ASVs	Proportion of core bacteria types	Relative abundance of core microbiota
High altitude	HH	1959	1105	56.41%	92.18%
	HM	2404		45.97%	89.16%
	HD	1965		56.23%	95.97%
Low altitude	LH	805	207	25.71%	73.39%
	LM	2063		10.03%	27.65%
	LD	878		23.58%	83.19%

Note: Proportion of core bacteria types: ratio of species of shared microbial (ASVs) to the total number of species detected; Relative abundance of core microbiota: total relative abundance of shared core ASVS in each species.

Table S3 Diversity and relative abundance of core microbiota between two groups

Altitude	Comparis on type	Groups	Number of cores ASVs	Proportion of bacteria types	Relative abundance of core microbiota
High altitude	HHvsHM	HH	1381	70.50%	93.42%
		HM		57.45%	90.85%
	HHvsHD	HH	1397	71.31%	97.41%
		HD		71.09%	97.97%
	HDvsHM	HD	1425	72.52%	97.13%
		HM		59.28%	94.76%
Low altitude	LHvsLM	LH	333	41.37%	76.05%
		LM		16.14%	29.53%
	LHvsLD	LH	348	43.23%	89.13%
		LD		39.64%	91.74%
	LDvsLM	LD	447	50.91%	89.65%
		LM		21.67%	78.70%

Note: Proportion of core bacteria types: ratio of species of shared microbial (ASVs) to the total number of species detected; Relative abundance of core microbiota: total relative abundance of shared core ASVS in each species.

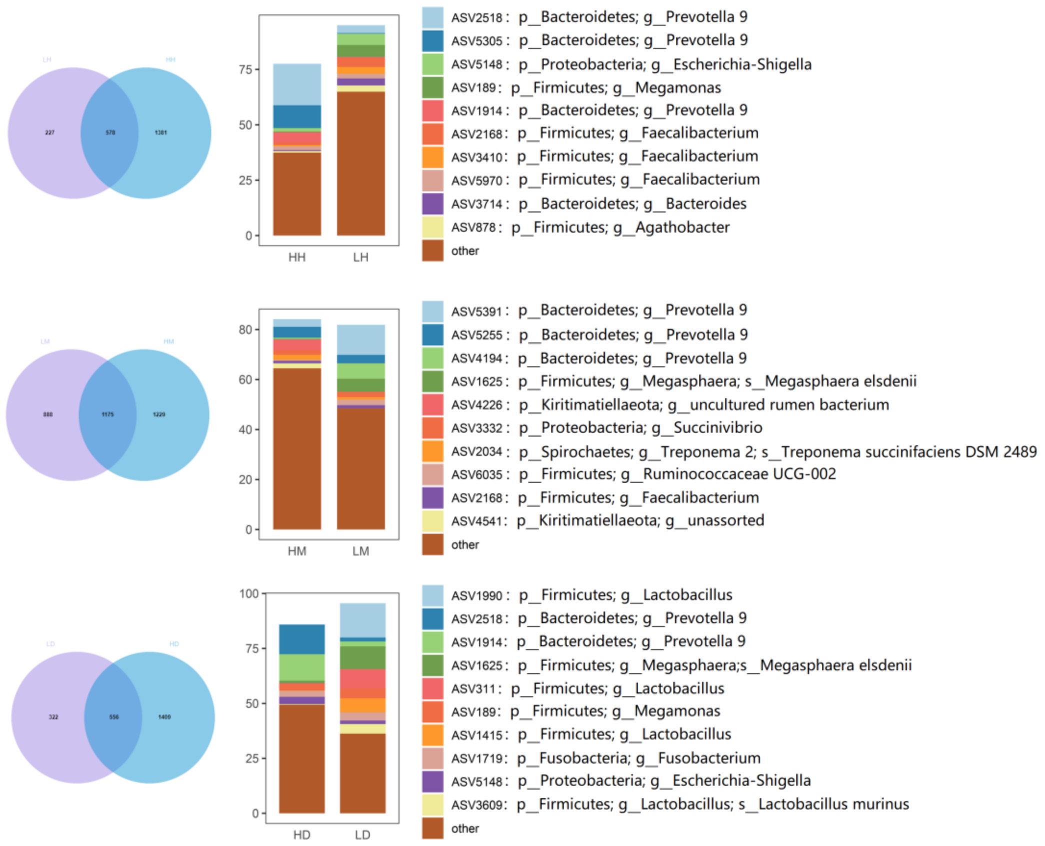

Figure. S1 Core ASVs in humans, monkeys, and dogs in high-altitude to low-altitude environments. Numbers in plots are marked for how many ASVs are in this part. The histogram represents the relative abundance of core ASVs. HH stands for high altitude humans, HD stands for high altitude domestic dogs, HM stands for high altitude wild macaques, LH stands for low altitude humans, LD stands for low altitude domestic dogs, LM stands for low altitude wild macaques.

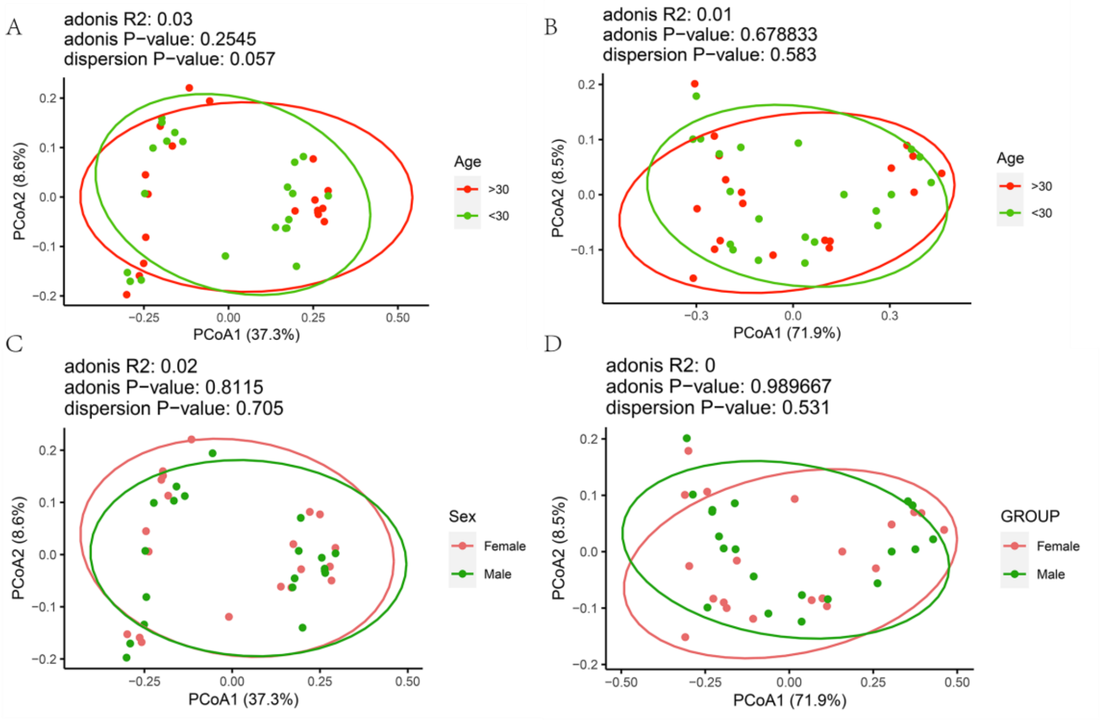

Figure. S2 Beta diversity analysis of microbial composition in different sexes and ages of humans. (A) The unweighted UniFrac distance based on PCoA plot is used to analyze the difference between aged 30 years and those younger than 30 years in humans. (B) The weighted UniFrac distance based on PCoA plot is used to analyze the difference between aged 30 years and those younger than 30 years in humans. (C) The unweighted UniFrac distance based on PCoA plot is used to analyze the difference between male and female in humans. (D) The weighted UniFrac distance based on PCoA plot is used to analyze the difference between male and female in humans
